# Temporal Patterns of Diabetes in Pregnancy: Analyzing Pregestational and Gestational Diabetes Mellitus Among Women Aged 15–44 Years Using the United States Diabetes Surveillance System (USDDS) Database

**DOI:** 10.7759/cureus.49694

**Published:** 2023-11-30

**Authors:** Elochukwu U Nwachukwu, Jennifer C Ezeamii, Tricia O Okoye, Okelue E Okobi, Rhoda O Ojo, Zainab Akinsola, Yonas H Gebeyehu, Ehireme A Osarenkhoe-Ighodaro

**Affiliations:** 1 Family Medicine, University of Uyo Teaching Hospital, Uyo, NGA; 2 Nursing Sciences, Faculty of Health Sciences and Technology, University of Nigeria Enugu Campus, Enugu, NGA; 3 Family Medicine, College of Medical Sciences, Ambrose Alli University, Ekopma, NGA; 4 Family Medicine, Larkin Community Hospital Palm Springs Campus, Hialeah, USA; 5 Family Medicine, Medficient Health Systems, Laurel, USA; 6 Family Medicine, Lakeside Medical Center, Belle Glade, USA; 7 Epidemiology and Biostatistics, University of Texas Health Science Center at Houston, Houston, USA; 8 Internal Medicine/Family Medicine, Windsor University School of Medicine, Cayon, KNA; 9 Medicine, Addis Ababa University, Addis Ababa, ETH; 10 Family Medicine, Garki Hospital Abuja, Abuja, NGA

**Keywords:** gestational diabetes mellitus, pregestational diabetes mellitus, diabetes in pregnancy, trend analysis, usdds

## Abstract

Background: Diabetes in pregnancy, including pregestational diabetes mellitus (PGDM) and gestational diabetes mellitus (GDM), is a significant global health concern. Understanding its temporal patterns is crucial for effective healthcare planning and intervention strategies.

Methods: This retrospective observational study utilized data from the United States Diabetes Surveillance System (USDDS) spanning 2000 to 2019. We conducted a comprehensive analysis of temporal trends in PGDM and GDM prevalence among people aged 15-44 years. Additionally, age and race-based subgroup analyses were performed to identify variations in diabetes patterns.

Results: Over the two-decade study period, PGDM and GDM exhibited distinct temporal patterns. PGDM prevalence remained stable initially (1.1% per 100 deliveries) but gradually increased to 1.6% by 2019. In contrast, GDM prevalence showed a consistent rise, reaching 9.6% per 100 deliveries by 2019. Age-specific analysis revealed higher prevalence rates in older age groups, peaking at 40-44 years. Race-based analyses unveiled significant disparities, with Asians having the highest GDM rates and Black individuals having the highest PGDM rates.

Conclusion: The prevalence of diabetes in pregnancy in the United States has increased significantly from 2000 to 2019, emphasizing the need for continued surveillance and tailored interventions. Age and race-specific disparities highlight the importance of the growing impact of diabetes in pregnancy on maternal and fetal health.

## Introduction

Diabetes mellitus, characterized by elevated blood glucose levels, is a global public health concern. Within this spectrum, diabetes during pregnancy, including pre-gestational diabetes (PGDM) and gestational diabetes mellitus (GDM), poses unique challenges. The prevalence of diabetes in pregnancy has been steadily increasing, mirroring the global diabetes surge, necessitating comprehensive investigation to understand trends, evaluate interventions, and guide healthcare strategies [1,2]. The epidemiology of diabetes in pregnancy, spanning PGDM and GDM, is of significant public health importance. In 2021, the International Diabetes Federation reported approximately 537 million people (20-79 years) living with diabetes worldwide, with a projected increase to 643 million by 2030 [3,4]. In the United States (US), diabetes, encompassing PGDM and GDM, is a major concern. In 2020, the Centers for Disease Control and Prevention (CDC) identified about 34.2 million people with diabetes, constituting approximately 10.5% of the US population, inclusive of undiagnosed cases. GDM prevalence varies, affecting 2-10% of pregnancies [5].

Diabetes in pregnancy arises from intricate interactions involving physiological changes, genetics, and environmental factors. Pre-existing PGDM includes type 1 or type 2 diabetes marked by insulin resistance and inadequate production. In type 1, pancreatic beta cell destruction causes insulin deficiency, while type 2 involves resistance and deficiency [6,7]. Hormonal shifts during pregnancy worsen insulin resistance, requiring increased pancreatic compensation. In PGDM, inadequate compensation leads to hyperglycemia, risking the mother and fetus. Conversely, GDM emerges later due to glucose tolerance issues, compounded by insulin antagonists. The resulting hyperglycemia poses risks such as stillbirth, preterm birth, and congenital anomalies. Long-term consequences include an elevated likelihood of metabolic and cardiovascular diseases, perpetuating the global diabetes cycle [8-10].

This study delves into the temporal patterns of diabetes in pregnancy from 2000 to 2019, with a specific focus on PGDM and GDM among a population aged 15-44 years, using a comprehensive dataset. This demographic range covers women of childbearing age, a critical period when pregnancy is more likely to occur. Understanding the prevalence and impact of diabetes in this age group is crucial for maternal and fetal health. This study uses data from the US Diabetes Surveillance System (USDDS) database, a reliable source of information on diabetes prevalence and related trends across the US, to understand how diabetes during pregnancy has changed in the last 20 years [11]. Temporal analysis of diabetes in pregnancy based on factors such as age, race, and time period holds several critical implications. Firstly, it offers insights into changing PGDM and GDM prevalence, informing resource allocation, preventive strategies, and targeted interventions. Secondly, it identifies high-risk periods and subpopulations, refining screening protocols, and tailoring care to pregnant individuals with diabetes. Lastly, it evaluates intervention effectiveness and policy impact, guiding future strategies for optimal maternal and neonatal health outcomes.

## Materials and methods

Study design and data source

In this retrospective observational study, our objective was to analyze the temporal patterns of diabetes in pregnancy among a population aged 15-44 years in the US from 2000 to 2019. We utilized data from the USDDS database, which served as a comprehensive repository of diabetes-related information. This database included a wealth of epidemiological data, such as diagnosis records, demographic information, clinical data, and geographic variables, making it a robust resource for investigating trends in diabetes in pregnancy over two decades. The study strictly adhered to all ethical guidelines and regulations concerning the use of de-identified health data.

Study population

The study comprised a substantial sample size, with a total of 4284 million cases of GDM and 696 million cases of PGDM. This extensive dataset covered a population aged 15-44 years in the US, spanning the years from 2000 to 2019. Pregnant individuals with these diabetes types constituted the core of the study population, and their data were extracted for analysis.

Variables of interest

The primary outcome variables of interest were the incidence and prevalence of PGDM and GDM among the study population. These were calculated based on the number of cases identified within specific time intervals, such as yearly, during the study period. The primary independent variables of interest encompassed age and race/ethnicity, enabling a comprehensive examination of temporal patterns in diagnosed diabetes cases across diverse demographic segments.

Data analysis

The data analysis commenced with an initial phase of descriptive statistics, which characterized the study population, including the prevalence of PGDM and GDM over time. Subsequently, we delved into the exploration of temporal patterns in PGDM and GDM through the application of statistical techniques, such as time series analysis and graphical representations (e.g., line charts). The Poisson regression model was used to obtain confidence intervals for the incidence rate. This analytical approach allowed us to assess whether there were significant increases, decreases, or fluctuations in diabetes prevalence during the study period. Furthermore, subgroup analyses were performed to investigate potential variations in temporal patterns by demographic factors, such as age, gender, and race/ethnicity.

## Results

The study analyzed a total of 4,284,799,000 cases of GDM and 696,438,000 cases of PGDM, encompassing the population aged 15-44 years in the US from 2000 to 2019. Overall, the analysis of the USDDS database revealed an increasing rate of temporal patterns in diagnosed diabetes cases. These patterns were characterized by variations based on age and race/ethnicity, providing valuable insights into the diabetes burden over the past two decades.

Among the diabetes cases, 1.20% (95%CI: 1.13-1.28) were diagnosed with PGDM while 7.22% (95%CI: 6.98-7.49) were diagnosed with GDM during the study period. The demographic characteristics of the study population are summarized in Table 1 and Table 2.

**Table 1:** PGDM rate per 100 deliveries among females aged 15-44 years ** Unavailable data PGDM: pregestational diabetes mellitus

	Pre-pregnancy Diabetes Mellitus	2000	2001	2002	2003	2004	2005	2006	2007	2008	2009	2010	2011	2012	2013	2014	2015	2016	2017	2018	2019	Total
	Total - Percentage	0.8	0.8	0.9	1	1.1	1	1.1	1.1	1.1	1.1	1.3	1.2	1.3	1.3	1.3	1.4	1.5	1.5	1.6	1.6	1.20
	Total - Lower Limit	0.7	0.7	0.9	0.9	1	0.9	1	1	1	1	1.2	1.1	1.3	1.3	1.3	1.4	1.4	1.4	1.5	1.5	1.13
	Total - Upper Limit	0.9	0.9	1	1.1	1.1	1.1	1.2	1.2	1.2	1.2	1.4	1.3	1.4	1.4	1.4	1.5	1.5	1.5	1.6	1.7	1.28
	Total - Number in 1000s	25134	25630	30161	32212	34607	33306	35847	38923	34718	35361	37604	35270	38395	38540	40350	**	43190	44005	46045	47140	696438.00
	Total - Lower Limit	22380	22865	26943	28158	31229	29516	32047	34686	30943	31911	34168	31558	36520	36678	38420	Suppressed	41111	41854	43801	44893	639681.00
	Total - Upper Limit	27889	28395	33378	36266	37985	37096	39646	43161	38493	38811	41040	38983	40270	40402	42280	Suppressed	45269	46156	48289	49387	753196.00
Age data	15-19 - Rate per 100 deliveries	0.2	0.3	0.3	0.3	0.3	0.3	0.3	0.3	0.3	0.3	0.4	0.3	0.4	0.4	0.4	0.5	0.5	0.5	0.5	0.6	0.37
	20-24 - Rate per 100 deliveries	0.4	0.4	0.5	0.5	0.6	0.5	0.5	0.6	0.6	0.5	0.6	0.6	0.6	0.6	0.7	0.7	0.7	0.7	0.8	0.8	0.60
	25-29 - Rate per 100 deliveries	0.6	0.6	0.7	0.8	0.8	0.8	0.8	0.8	0.8	0.8	0.9	0.8	0.9	0.9	0.9	0.9	0.9	0.9	1	1.1	0.84
	30-34 - Rate per 100 deliveries	0.8	0.8	0.9	1	1	1	1.1	1.1	1	1.1	1.2	1.2	1.2	1.2	1.2	1.3	1.3	1.3	1.3	1.4	1.12
	35-39 - Rate per 100 deliveries	1	1.2	1.3	1.4	1.4	1.4	1.5	1.5	1.4	1.6	1.9	1.7	1.8	1.9	1.9	1.9	2	2.1	2	2.1	1.65
	40-44 - Rate per 100 deliveries	1.5	1.3	1.8	1.8	2	1.8	2.2	2.2	2.1	2	2.5	2.5	2.6	2.7	2.7	2.9	2.9	3	3.2	3.2	2.35
Race	Hispanic - Rate per 100 deliveries	**	**	**	**	**	**	**	**	**	**	**	**	1.7	1.9	1.8	1.9	1.9	2	2.1	2.1	1.93
	Non-Hispanic White - Rate per 100 deliveries	**	**	**	**	**	**	**	**	**	**	**	**	1	1	1.1	1	1	1.1	1.2	1.2	1.08
	Non-Hispanic Black - Rate per 100 deliveries	**	**	**	**	**	**	**	**	**	**	**	**	2.1	2.3	2.2	2.3	2.4	2.4	2.3	2.5	2.31
	Non-Hispanic Asian or Pacific Islander - Rate per 100 deliveries	**	**	**	**	**	**	**	**	**	**	**	**	1	1	1	1.4	1.2	1.2	1.4	1.5	1.21

**Table 2:** GDM rate per 100 deliveries among females aged 15-44 years ** Unavailable data GDM: gestational diabetes mellitus

	Gestational Diabetes Mellitus	2000	2001	2002	2003	2004	2005	2006	2007	2008	2009	2010	2011	2012	2013	2014	2015	2016	2017	2018	2019	Total
	Total - Percentage	4.5	5.2	5.5	5.4	5.8	5.9	6.7	6.9	6.9	7	7.2	7.7	8.2	8.1	8.3	8.3	8.6	9.1	9.5	9.6	7.22
	Total - Lower Limit	4.3	5	5.2	5.2	5.6	5.6	6.4	6.5	6.5	6.7	6.9	7.4	8	7.9	8.1	8.1	8.5	8.9	9.3	9.4	6.98
	Total - Upper Limit	4.8	5.5	5.8	5.7	6.1	6.1	7	7.2	7.2	7.4	7.5	8.1	8.4	8.3	8.5	8.5	8.8	9.3	9.7	9.8	7.49
	Total - Number in 1000s	139466	158035	174954	165389	187256	188164	216839	240096	221632	219111	211675	229842	248750	250580	263165	**	274445	291255	300835	303310	4284799.00
	Total - Lower Limit	126737	145125	159208	152188	170907	171870	197910	218255	202362	199413	194017	208198	238805	240719	252414	Suppressed	263723	279701	288678	291023	4001253.00
	Total - Upper Limit	152195	170946	190700	178589	203605	204458	235767	261936	240903	238809	229334	251486	258695	260441	273916	Suppressed	285167	302808	312992	315597	4568344.00
Age data	15-19 - Rate per 100 deliveries	0.9	1.1	1.2	1.2	1.2	1.2	1.4	1.5	1.5	1.5	1.5	1.7	2	2	2.1	2.3	2.2	2.5	2.4	2.6	1.70
	20-24 - Rate per 100 deliveries	2	2.2	2.3	2.2	2.3	2.4	2.8	2.9	3	3	3	3.2	3.5	3.5	3.6	3.6	3.8	4.1	4.5	4.5	3.12
	25-29 - Rate per 100 deliveries	3.5	3.9	4.2	4	4.3	4.3	4.9	5.1	5.1	5.1	5.2	5.6	6	5.9	6.1	6.1	6.2	6.5	6.9	7.2	5.31
	30-34 - Rate per 100 deliveries	4.7	5.5	5.8	5.5	6.1	6.3	7.3	7.4	7.3	7.4	7.5	8	8.3	8.3	8.5	8.5	8.8	9.4	9.6	9.9	7.51
	35-39 - Rate per 100 deliveries	6.4	7.5	8	7.9	8.2	8.4	9.7	10.1	9.9	10.1	10.7	11.6	11.9	11.8	12	11.9	12.3	13.1	13.5	13.6	10.43
	40-44 - Rate per 100 deliveries	8.7	10	10.2	10.5	11.3	11.2	12.7	12.5	12.8	13.4	13.6	14.4	15.2	15	15.5	15.5	16.4	17	18.1	17.8	13.59
Race	Hispanic - Rate per 100 deliveries	**	**	**	**	**	**	**	**	**	**	**	**	10.8	10.4	10.5	10.8	10.9	11.3	11.9	12.1	11.09
	Non-Hispanic White - Rate per 100 deliveries	**	**	**	**	**	**	**	**	**	**	**	**	6.8	6.9	6.9	7	7.3	7.8	8.1	8.2	7.38
	Non-Hispanic Black - Rate per 100 deliveries	**	**	**	**	**	**	**	**	**	**	**	**	7.3	7	7.6	7.2	7.4	7.8	7.9	7.9	7.51
	Non-Hispanic Asian or Pacific Islander - Rate per 100 deliveries	**	**	**	**	**	**	**	**	**	**	**	**	11.9	11.9	12.8	12.6	12.4	13.6	14.9	15.1	13.15

Overall trends

Notable temporal patterns could be seen in the rate of diabetes in pregnancy over the study period. Tables 1, 2 illustrate these trends, displaying changes in the overall prevalence of PGDM and GDM per 100 deliveries in the age group of 15-44 years. During the initial years of the study (2003-2009), the prevalence of PGDM remained relatively stable at 1.1% per 100 deliveries, while GDM exhibited a gradual increase, from 5.3% to 7.0% per 100 deliveries during the same period. Subsequently, from 2010 to 2018, both PGDM and GDM experienced a steady rise. PGDM prevalence increased to 1.6% per 100 deliveries and GDM prevalence reached 9.5% per 100 deliveries. By the end of the study period in 2019, PGDM prevalence increased to 1.6% per 100 deliveries, while GDM prevalence reached 9.6% per 100 deliveries.

Age-based analysis

The study conducted a subgroup analysis based on age to explore variations in temporal patterns of diabetes in pregnancy. In the 15-19 age group, the rate of diabetes cases per 100 deliveries was 1.7% for GDM and 0.37% for PGDM over the study period. This age group exhibited a consistent increase in the rate over the years in both groups except in 2011 (0.3% compared to 0.4% in 2010). Among those aged 20-24 years, the rate of diabetes cases per 100 deliveries was 3.12% for GDM and 0.60% for PGDM during the study period. Temporal patterns in this age group indicated a consistent increase in the rate over the years in both groups.

In the age group of 25-29 years, the rate of diabetes cases per 100 deliveries stood at 5.31% for GDM and 0.84% for PGDM over the study period. Temporal patterns in this group displayed increasing trends throughout the study period. Among those aged 30-34 years, the rate of diabetes cases per 100 deliveries was 7.51% for GDM and 1.12% for PGDM during the study period. Temporal patterns within this age group demonstrated fluctuations in GDM and a consistent increase in PGDM over the years.

**Figure 1:**
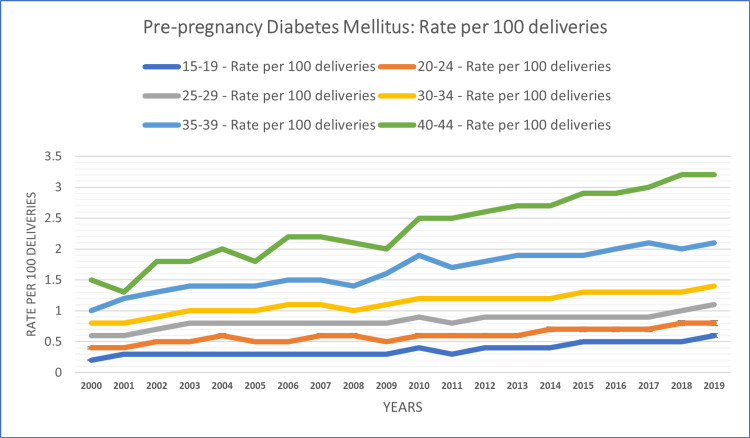
Age-based patterns of PGDM rate per 100 deliveries among females aged 15-44 years PGDM: pregestational diabetes mellitus

**Figure 2:**
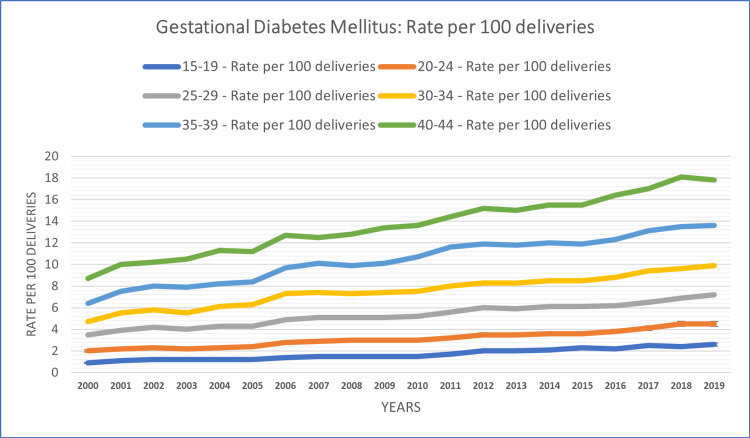
Age-based patterns of GDM rate per 100 deliveries among females aged 15-44 years GDM: gestational diabetes mellitus

In the age group of 35-39 years, the rate of diabetes cases per 100 deliveries reached 10.43% for GDM and 1.65% for PGDM over the study period. Temporal patterns observed in this cohort indicated fluctuations over the years in both groups. Among those aged 40-44 years, the rate of diabetes cases per 100 deliveries was 13.59% for GDM and 2.35% for PGDM during the study period. Temporal patterns within this age group showed fluctuations in the early years and a consistent increasing trend in later years. The age group-based analysis of the study population is summarized in Figures 1, 2.

Race/ethnicity-based analysis

**Figure 3:**
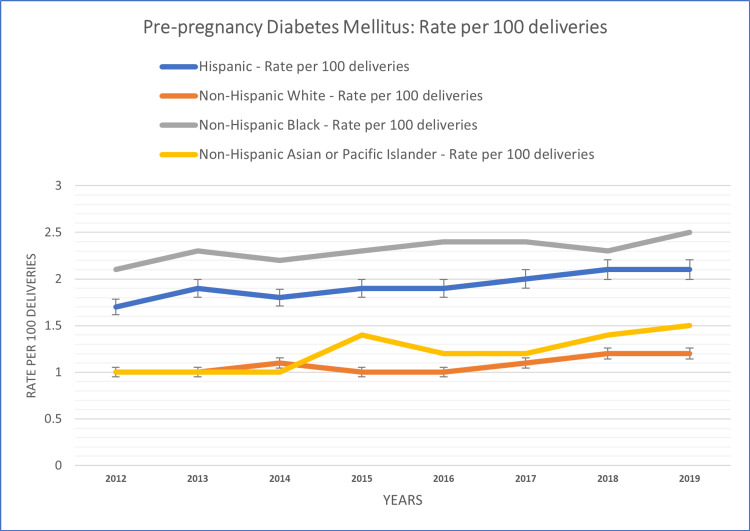
Race/ethnicity-based patterns of PGDM rate per 100 deliveries among females aged 15-44 years PGDM: gestational diabetes mellitus

**Figure 4:**
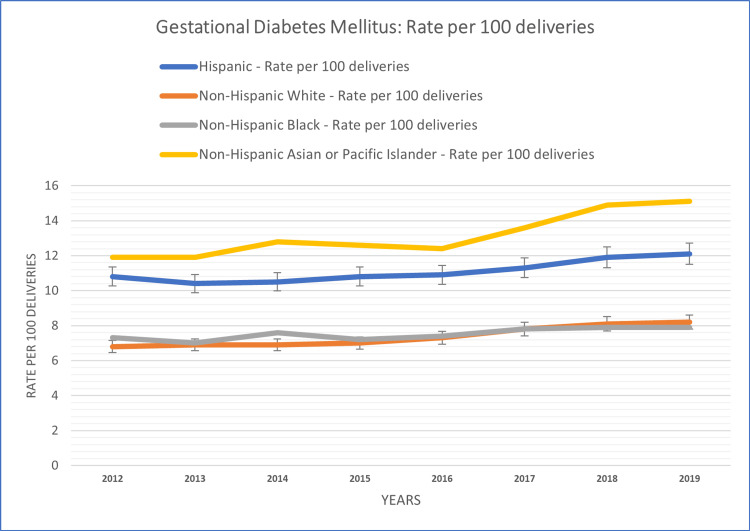
Race/ethnicity-based patterns of GDM rate per 100 deliveries among females aged 15-44 years GDM: gestational diabetes mellitus

The study also conducted subgroup analyses based on race and ethnicity to examine variations in the temporal patterns of diabetes in pregnancy. These race-based subgroup analyses revealed distinct temporal patterns of diabetes in pregnancy within different racial groups. The racial group-based analysis of the study population is summarized in Figures 3, 4.

Notably, the Asian population exhibited the highest rate per 100 deliveries for GDM prevalence at 13.15% over the study period. Following closely were the Hispanic population at 11.1%, the Black population at 7.51%, and the White population at 7.38%. These findings highlight significant variation in GDM prevalence among these racial groups during the study period. Conversely, PGDM displayed a different pattern, with the highest rate per 100 deliveries reported among the Black population at 2.31%. The Hispanic population followed with a rate of 1.93%, while the Asian population had a rate of 1.21%, and the White population had the lowest rate at 1.08%. These observations underscore the contrasting trends in PGDM prevalence among these racial groups during the same study period.

In the non-Hispanic White population, both PGDM and GDM prevalence were characterized by fluctuations. A consistent increase in the rate was observed in GDM (6.8% per 100 deliveries in 2012, 8.2% per 100 deliveries in 2019) and PGDM (1.0% per 100 deliveries in 2012, 1.2% per 100 deliveries in 2019) populations throughout the study period.

Among the non-Hispanic Black population, the temporal patterns of PGDM and GDM were characterized by fluctuations. PGDM prevalence showed an increasing trend over the years, with fluctuations, except for a slight reduction in 2018 (2.3% per 100 deliveries). GDM prevalence exhibited variations with some declining trends in 2013 (7.0% per 100 deliveries compared to 7.3% in 2012) and 2015 (7.2% per 100 deliveries compared to 7.6% in 2014).

The Hispanic population displayed distinct temporal patterns. The incidence and prevalence of GDM showed a substantial increase over the study period, without declines, except in 2013 (10.4% per 100 deliveries compared to 10.8% in 2012). PGDM prevalence displayed a consistent increase over the years, except for a slight decline in 2014 (1.8% per 100 deliveries compared to 1.9% in 2013).

Among the non-Hispanic Asian population, the temporal patterns of PGDM and GDM were characterized by fluctuations. GDM prevalence showed an increasing trend over the years, except for declines in 2015 (12.6% per 100 deliveries) and 2016 (12.4% per 100 deliveries). PGDM prevalence exhibited variations with declining trends in 2016 (1.2% per 100 deliveries).

## Discussion

Diabetes in pregnancy, encompassing both PGDM and GDM, presents a growing public health concern with profound implications for maternal and fetal health. The current study reveals dynamic temporal patterns in the prevalence of PGDM and GDM from 2000 to 2019. The rise in GDM prevalence, reaching 9.6% per 100 deliveries by 2019, is particularly noteworthy. This finding is consistent with global trends showing an increasing burden of GDM, likely attributed to factors such as rising obesity rates, sedentary lifestyles, and changes in diagnostic criteria, such as those outlined by the International Association of Diabetes and Pregnancy Study Groups (IADPSG) or the American College of Obstetricians and Gynecologists (ACOG) [12,13]. The significant increase in GDM prevalence underscores the importance of early detection and intervention, as GDM poses risks not only during pregnancy but also for the long-term health of both mother and child [12].

In contrast, PGDM prevalence remained the same, fluctuating between 1.1% and 1.6% per 100 deliveries over the study period. This observation aligns with the well-documented rise in type 2 diabetes worldwide [14]. While PGDM prevalence may not have surged as dramatically as GDM, it continues to be a significant concern due to the unique challenges it presents during pregnancy. Effective preconception care and management of PGDM are critical to mitigating adverse maternal and neonatal outcomes [15].

The age-based analysis in the current study revealed distinct patterns in diabetes prevalence across different age groups. Younger individuals aged 15-19 exhibited lower rates of both GDM and PGDM, while prevalence increased with advancing age. Advanced maternal age (usually defined as 35 years or older) is generally recognized as a risk factor for GDM and may prompt earlier and more intensive screening. Younger maternal age may also be considered in the IADPSG guidelines, as both extremes of age can be associated with an increased risk of diabetes during pregnancy. This finding is consistent with existing literature, which suggests that advanced maternal age is a risk factor for GDM [16,17]. The age-specific variations emphasize the need for tailored interventions and screening strategies, especially for older pregnant individuals.

Race and ethnicity-based analyses unveiled striking disparities in diabetes prevalence. The Asian population exhibited the highest rate of GDM prevalence, followed by the Hispanic, Black, and White populations. Conversely, the Black population had the highest PGDM prevalence, with the Hispanic, Asian, and White populations following suit. These disparities highlight the multifactorial nature of diabetes in pregnancy, influenced by genetic, socioeconomic, and lifestyle factors [18,19]. These findings underscore the importance of culturally sensitive and personalized approaches to diabetes care during pregnancy. Tailored interventions, early screening, and targeted educational programs can help address these disparities and improve outcomes for high-risk populations.

Understanding the temporal patterns of diabetes in pregnancy is crucial for healthcare policy and practice. Firstly, the rising trend in GDM prevalence calls for comprehensive screening programs and preventive measures. Early diagnosis and appropriate management can mitigate the associated risks, including macrosomia, preeclampsia, and neonatal hypoglycemia [20]. Secondly, age-specific care and monitoring should be integrated into prenatal services to address the varying prevalence of diabetes in different age groups. Older pregnant individuals should receive heightened attention to prevent and manage GDM effectively [17,21]. Lastly, reducing disparities among racial and ethnic groups is imperative. Health systems must prioritize equitable access to care, culturally competent healthcare providers, and community-based interventions to bridge the gaps in diabetes outcomes [19,22].

This study also possesses certain limitations. Firstly, the study does not account for potential changes in diagnostic criteria for diabetes and gestational diabetes over the study period. The accuracy of the findings of the present study may be influenced by modifications in guidelines and thresholds over time. Failure to address these changes could potentially limit the precision and reliability of the observed temporal patterns. It is crucial to consider and account for these evolving standards to ensure a more accurate interpretation of the study results and their relevance in the context of changing healthcare guidelines. A notable limitation of the study is that it did not assess key risk factors like previous GDM, BMI, and comorbid diseases, hindering its ability to thoroughly explain observed changes and limiting its power to understand the root causes of reported variations. Additionally, while the dataset is nationally representative, the study's findings may not fully extend to populations outside the US or to specific subpopulations with distinct healthcare systems and demographics. Understanding the factors driving the observed patterns would require further research and experimental studies.

The current study possesses several notable strengths. Firstly, it conducts a longitudinal analysis covering two decades, from 2000 to 2019, providing a perspective on the temporal patterns of diabetes in pregnancy. This extended timeframe allows for the identification of long-term trends and changes, contributing to a deeper understanding of the disease's evolution over time. Secondly, the study benefits from a large and nationally representative dataset, sourced from the USDDS database. This extensive and diverse data source enhances the reliability and generalizability of the findings, ensuring that the results are applicable to a broad spectrum of the US population. Furthermore, the inclusion of age and race-based analyses adds depth to the research. These demographic insights shed light on how diabetes in pregnancy affects different groups within the population. Such granularity is essential for tailoring interventions and healthcare strategies to specific subpopulations, ultimately improving healthcare outcomes.

## Conclusions

The extensive two-decade analysis of diabetes in pregnancy, utilizing the robust USDDS database, provides valuable insights into the evolving landscape of PGDM and GDM in the US. The findings highlight dynamic temporal patterns, revealing increasing prevalence rates for both PGDM (0.8% in 2000 to 1.6% in 2020) and GDM (4.5% in 2000 to 9.6% in 2020) over the study period. This underscores the urgency of continued monitoring and proactive healthcare strategies to address the growing burden of diabetes in pregnancy. Age-based analyses demonstrate varying prevalence rates across different age groups, emphasizing the need for age-specific interventions and screening protocols. Additionally, race and ethnicity-based disparities in diabetes prevalence underscore the importance of tailored healthcare approaches to address the unique needs of diverse populations. It serves as a vital foundation for future research and the development of targeted interventions to mitigate the impact of diabetes in pregnancy on maternal and fetal health. As healthcare continues to evolve, addressing the challenges posed by diabetes in pregnancy remains a critical public health priority.
